# Itch and Pain Behaviors in Irritant Contact Dermatitis Produced by Sodium Lauryl Sulfate in Mice

**DOI:** 10.3390/ijms25147718

**Published:** 2024-07-14

**Authors:** Nathalie M. Malewicz-Oeck, Zhe Zhang, Steven G. Shimada, Robert H. LaMotte

**Affiliations:** 1 Clinics for Anesthesiology, Intensive Care and Pain Medicine, University Hospital of Ruhr University Bergmannsheil Bochum, 44789 Bochum, Germany; 2 Department of Anesthesiology, Yale University School of Medicine, 330 Cedar St., New Haven, CT 06510, USA

**Keywords:** irritant contact dermatitis, itch, pain, hyperalgesia, inflammation, SLS

## Abstract

Irritant contact dermatitis (ICD) is a nonspecific skin inflammation caused by irritants, leading to itch and pain. We tested whether differential responses to histamine-dependent and -independent pruritogens can be evoked in ICD induced by sodium lauryl sulfate (SLS). An ICD mouse model was established with 5% SLS in acetone versus a vehicle topically applied for 24 h to the cheek. Site-directed itch- and pain-like behaviors, occurring spontaneously and in response to mechanical, thermal, and chemical stimuli (histamine, ß-alanine, BAM8-22, and bradykinin) applied to the cheek, were recorded before (day 0) and after irritant removal (days 1, 2, 3, and 4). Skin inflammation was assessed through visual scoring, ultrasound, and measurements of skin thickness. SLS-treated mice exhibited hyperalgesia-like behavior in response to mechanical and heat stimuli on day 1 compared to the controls. SLS mice exhibited more spontaneous wipes (pain) but not scratching bouts (itch) on day 1. Pruritogen injections caused more scratching but not wiping in SLS-treated mice compared to the controls. Only bradykinin increased wiping behavior compared to saline. SLS-treated mice developed noticeable erythema, scaling, and increased skin thickness on days 1 and 2. SLS induced cutaneous inflammation and behavioral signs of spontaneous pain and itching, hyperalgesia to mechanical and heat stimuli and a chemical algogen, and enhanced itch response to pruritogens. These sensory reactions preceded the inflammation peak and lasted up to two days.

## 1. Introduction

Contact dermatitis is an inflammatory reaction of the skin to a substance that either irritates the skin (irritant contact dermatitis (ICD)) or triggers an allergic response (allergic contact dermatitis (ACD)) after repeated exposure [1]. ICD is a nonimmunologic and nonspecific inflammatory response to direct cutaneous injury caused by contact with an irritating substance [2,3]. Symptoms characterizing a severe skin reaction include erythema (redness), edema (swelling), vesiculation (fluid-filled bumps or blisters), and the development of scaling, crusting, and cracks or fissures. Sensory symptoms include burning and/or itching and hypersensitivity (enhanced itching or pain) to certain mechanical or thermal cutaneous stimuli.

In humans, ICD represents up to 7% of all dermatological cases and 80% of contact dermatitis-associated occupational skin disorders [4,5,6]. The development of ICD is caused by exposure to an irritant, leading to a disrupted skin barrier and increased penetration of irritants [7]. This triggers an inflammatory response with dysregulation of proinflammatory cytokines and chemokines [7,8,9] and immunologic activation [10]. The clinical manifestation of ICD can vary significantly based on several factors, including the strength, amount, duration, frequency, and concentration of irritants, as well as environmental factors and underlying skin conditions [4]. Therefore, one challenge posed by ICD lies in its heterogeneous nature. It encompasses a spectrum of conditions with diverse clinical manifestations and underlying causes. Examples include acute, delayed acute, and cumulative (chronic) ICD, each presenting unique challenges in both clinical treatment and experimental investigation [4,5]. In the treatment of ICD, avoidance of irritants is a major factor, and moisturizers are commonly employed to enhance the regeneration of the skin barrier compared to untreated irritated skin [7]. 

Sodium lauryl sulfate (SLS, or sodium dodecyl sulfate (SDS)) is an irritant, anionic, surface-active detergent that causes ICD. It is commonly applied as an emulsifier in cosmetics (soaps), detergents, foods (oils), and pharmaceutical vehicles and therefore has a wide application [7]. It is utilized for studying cutaneous irritation in humans and animals [11]. Among the various irritants used to induce experimental ICD models, SLS stands out due to its ability to disrupt the skin barrier and trigger inflammatory responses similar to those observed in clinical settings [8]. To accurately compare models and clinical settings, it is essential to assess symptomology [12]; consequently, both sensory analyses in humans and fitting valid behavioral analyses in rodents must be applied. Behavioral analysis to assess the effects of raw cosmetic or cleanser products is rarely the focus of such studies [13], though these products could potentially lead to detrimental effects, as in the case of SLS inducing ICD. Considering that chemicals in washing and cleansing products are often applied to the face, it is crucial to investigate these in combination with other chemicals and factors, as real-life scenarios involve co-application and mechanical and thermal stimulation [14]. Moreover, variations in skin sensitivity and recovery rates depending on the irritated anatomical region underscore the need for standardized testing protocols when assessing the effectiveness of treatments [12,15,16]. Therefore, a uniformly applied model and protocols are of high importance.

Inducing painful and pruritic models on the cheeks of mice offers a valid system for investigating acute and chronic itch and pain through behavioral analysis [1,17,18]. This approach allows for the inclusion of responses to injected chemicals and pruritogens in models that induce itch and pain through different pathways to differentiate underlying mechanisms and pathology [19,20,21]. We have recently established mechanical and thermal testing protocols specifically for the cheek [22]. For example, in a model of ACD on the cheek, mice exhibited enhanced itch-like scratching in response to pruritogens and an algogen, which typically elicited only pain-like wiping behavior of normal skin without inducing scratching [23]. This approach enables more precise and comprehensive investigation of models like the SLS-induced ICD model and better representation of clinical settings. 

This study aimed to determine whether ICD produced by SLS elicited excessive site-directed scratching and/or wiping behaviors occurring spontaneously or in response to chemical, mechanical, or thermal stimuli.

## 2. Results

### 2.1. Behavioral Responses to Mechanical Stimulation

**Figure 1:**
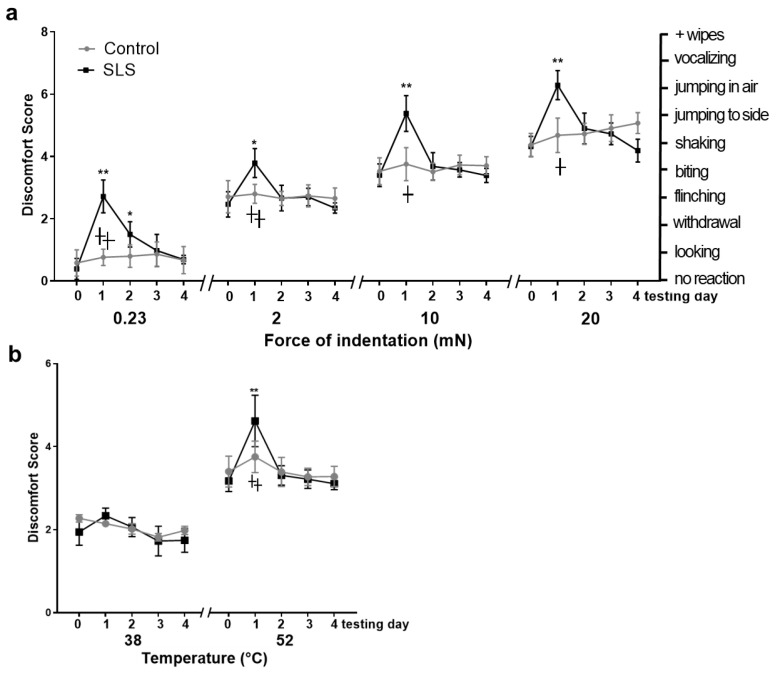
Effects of SLS-induced ICD on DS derived in response to vFFs and a heated contact thermode placed to the cheek. Stimuli were applied before (day 0) and 1 h (day 1), 24 h (day 2), 48 h (day 3), and 72 h (day 4) after SLS or vehicle application. The mean DSs are shown in black for SLS and grey for the control group, respectively. (**a**) Mean DSs ± SDs for an innocuous vFF of 0.23 mN and mean DSs for a noxious vFF with a 100 µm tip diameter applied with three different bending forces (2, 10, and 20 mN). (**b**) Mean DSs ± SDs for innocuous (38 °C) and noxious t (52 °C) temperature stimuli. Significant differences before vs. after application for 24 h within groups are marked as follows: * *p* < 0.05, ** *p* < 0.01. Significant differences between the groups are marked as follows: † *p* < 0.05, †† *p* < 0.01. Data: means ± SDs. N = 12 mice/group.

After mechanical stimulation, differences in the mean DSs (discomfort scores) revealed significant main effects of treatment, force, and time of testing and an interaction between treatment, group, time, and force. The mean DSs significantly increased with bending forces for both the control and SLS groups on each day after the irritant removal. The control mice exhibited no consistent change in mean DS for both mechanical stimuli across all days. Compared with day 0, the mean DS in the SLS group was significantly greater on day 1 as a reaction to an innocuous vFF (von Frey filament) applied with a 0.23 mN force (allodynia) and to each of the typically aversive vFFs applied with forces of 2 to 20 mN (hyperalgesia). The SLS-group mice had a significantly greater mean DS than the control mice in response to each filament (Figure 1a), but only on day 1. No differences between groups were found on subsequent days for any filament.

### 2.2. Behavioral Responses to Heat Stimulation

Significant main effects of treatment, temperature, and day of testing were found in response to thermal stimulation. For both the control and SLS groups, before and each day after the irritant removal, the mean DS was higher after 52 °C compared to 38 °C. For the innocuous temperature of 38 °C, no change in the mean DS was observed in the control or SLS groups on any day. However, after noxious heat of 52 °C, a significant difference in the mean DS was only found on days 1 and 2 (hyperalgesia) compared with day 0 in the SLS group and in a comparison of the SLS and the control group (Figure 1b).

### 2.3. Spontaneous Behavior

**Figure 2:**
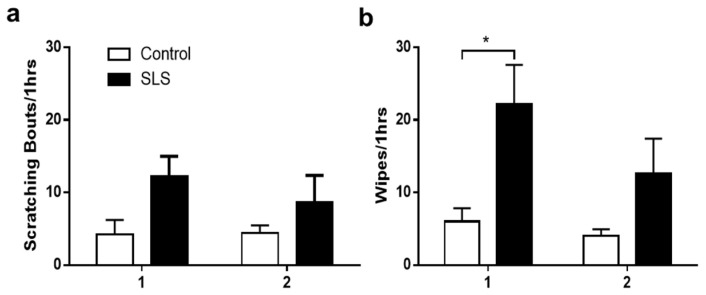
Effects of SLS-induced ICD on spontaneous scratching and wiping behaviors. Spontaneous scratching bouts (**a**) and wipes (**b**) were counted 1 h (day 1) and 24 h (day 2) after removal of the distilled water (control group) or 5% SLS (SLS group). * *p* < 0.05 indicates significance between groups. Data: means ± SEMs. N = 12 mice/group.

ICD mice exhibited an increased mean number of spontaneous wipes but not scratching bouts on days 1 and 2 in comparison to the control mice (Figure 2). No differences were significant between days 1 and 2 for either the SLS or the control groups. 

### 2.4. Behavioral Responses to Pruritogens and an Algogen

**Figure 3:**
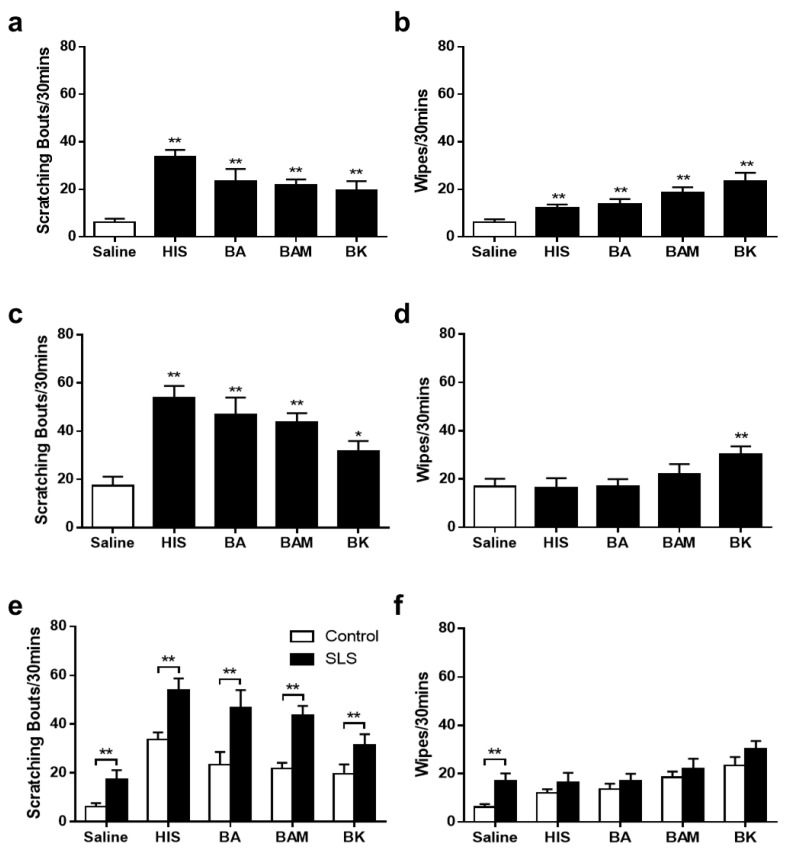
Effects of SLS on scratching and wiping evoked by intradermal injection of saline, bradykinin, and each pruritogen. Chemicals were injected into cheek skin after a 24 h treatment with either distilled water (control group) or 5% SLS (SLS group). (**a**,**b**) Mean numbers of scratching bouts (**a**) and wipes (**b**) evoked by the intradermal injection of each chemical into skin of the cheek previously treated with distilled water. (**c**,**d**) Mean numbers of scratching bouts (**c**) and wipes (**d**) evoked by an injection (intradermally) of each chemical into the cheek skin area previously treated with 5% SLS. (**e**,**f**) Comparison of the effects of SLS vs. distilled water on the mean numbers of scratching bouts (**e**) and wipes (**f**) evoked by single chemical injection. Significant differences for comparison with saline are marked as follows: * *p* < 0.05, ** *p* < 0.01. Significant differences in panels (**e**,**f**) for comparison between the control group and the SLS group are marked as follows: ** *p* < 0.01. Error bars: SEMs. N = 12 mice/group.

When responses to an injection of saline were compared, the control mice exhibited significantly more bouts of scratching and wipes in response to both the algogen bradykinin and the pruritogens histamine, BA, and BAM8-22 (Figure 3a,b). After SLS application, significantly more scratching was evoked by each pruritogen and the algogen in comparison with saline (Figure 3c). Only BK led to significantly higher wiping numbers in comparison to saline injection (Figure 3d). SLS-treated mice exhibited more scratching but not wiping in comparison to the control mice (Figure 3e,f). However, they also showed significantly more scratching in response to saline. This increased response to saline may have contributed to the greater response to each pruritogen and bradykinin. 

### 2.5. Severity of Inflammation of Cheek Skin

**Figure 4:**
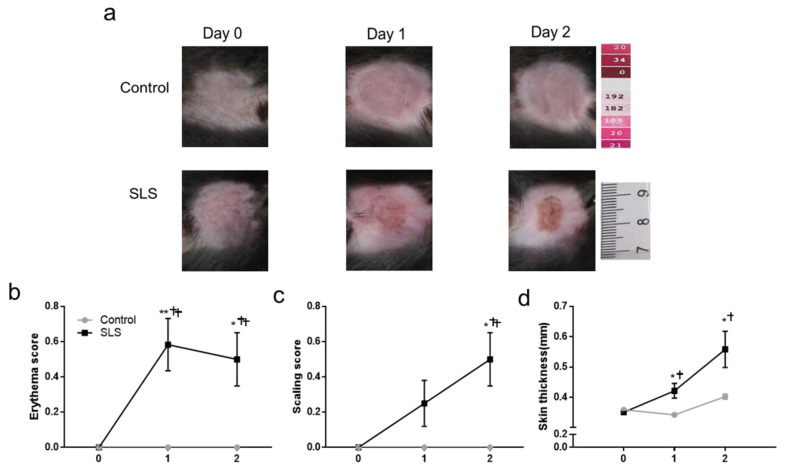
Effects of SLS-induced ICD on the severity of inflammation of cheek skin. (**a**) Exemplary photographs of the skin of the cheek for each experimental condition and group (SLS group vs. control group) before and each day after removing the PEEK cup showing a noticeable change in erythema and scaling. Erythema (**b**) and scaling (**c**) were separately evaluated on a scale from 0 to 4, corresponding to “none” to “very marked”. The thickness of a fold of cheek skin (**d**) was obtained with a micrometer. Significant differences for comparison between days are marked as follows: * *p* < 0.05, ** *p* < 0.01. Significant differences between the groups (SLS versus control) are marked as follows: † *p* < 0.05, †† *p* < 0.01. Error bars: SEMs. N = 12 mice/group.

Cheek skin was photographed on each day in each group. For all the groups, after treatment, a roundly shaped area of hair removal surrounding the treatment area was left by the removal of the PEEK cups. The control mice showed no indications of redness, scaling, or swelling at all time points. In contrast, SLS led to an erythema of the skin on day 1 and day 2, and a slight scaling was seen on day 1 and was more pronounced on day 2 (Figure 4a). 

The erythema and scaling were scored, and skin-fold thickness was measured. The control mice had no erythema, scaling, nor increased thickness of the skin after treatment with distilled water. The erythema score and skin thickness showed an increase on day 1 and day 2 in the SLS group, whereas the scaling score increased significantly only on day 2 (Figure 4b–d). In comparison with the control group, SLS mice had significantly higher values of erythema and skin thickness on both day 1 and day 2, but a significantly higher scaling score only on day 2 (Figure 4b–d). 

### 2.6. High-Frequency Ultrasound Imaging of Skin Layers

**Figure 5:**
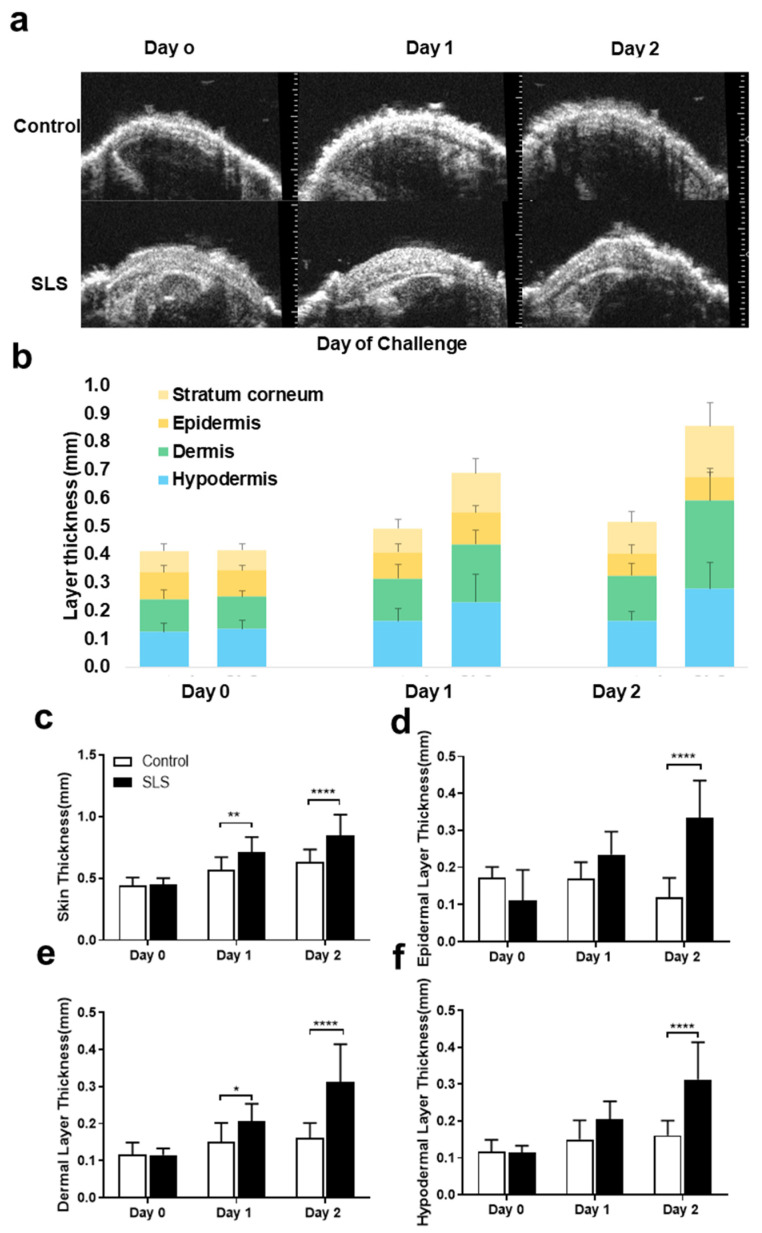
Effects of SLS-induced ICD on cheek skin assessed with ultrasound. (**a**) Exemplary ultrasound skin images (**a**) were used to measure the changes in different skin layers in SLS and control groups over time (**b**). Skin thickness measurements obtained from ultrasound images (**c**) and thickness measurements of different layers of the epidermis (**d**), dermis (**e**), and hypodermis (**f**). Significant differences are marked as follows: * *p* < 0.05, ** *p* < 0.01, **** *p* < 0.0001. N = 10–12 mice/group.

Ultrasound images of each mouse’s cheek were acquired on each day (day 0, day 1, and day 2) for each group and condition. Exemplary ultrasound images for the control group showed similar features, such as skin thickness and smoothness, across all days. There was no difference displayed in overall skin thickness or each skin-layer thickness between each day in the control group and day 0 in the different groups. The SLS group showed an increased overall skin thickness on day 1—more so on day 2 (Figure 5c). This was also indicated by an increase in the thickness of each layer on day 2 (Figure 5d–f). The highest increase was seen in the epidermis and dermis, especially on the second day. The increase in dermal thickness even reached significance on the first day.

## 3. Discussion

In this study, we successfully developed an ICD model using 5% SLS dissolved in acetone applied consistently to the cheeks of mice using occlusion via PEEK cups. This approach allowed precise administration of SLS and facilitated rapid induction of the model, reducing suffering and pain. 

SLS-induced ICD increased pain-like spontaneous behavior. In contrast, SLS did not increase the incidence of itch-like spontaneous behavior. These results are consistent with previous findings in humans that SLS-treated skin was accompanied by inflammatory pain sensation but little or no spontaneous itching [24], and, in one study in mice, no spontaneous scratching was observed [24,25,26]. However, in a clinical setting, most forms of ICD are accompanied by frequent pruritus [7]. Therefore, the SLS-induced ICD model in mice may not be an accurate representation for studying spontaneous itch. 

Symptomology and behavioral analysis are important aspects in assessing murine models. In assessing the side effects of cosmetic raw products such as triclosan, an antibacterial compound used in many cosmetic products, other authors have uncovered behavioral impairments [27].

We also found that SLS mice exhibited allodynia and hyperalgesia on day 1 after punctate mechanical stimuli and hyperalgesia to heat. These findings are consistent with those obtained in humans, where 1% and 2% SLS produced temporary hyperalgesia to heat and mechanical stimuli that disappeared within 2–3 days [24]. This finding aligns with previous studies highlighting the temporal dynamics of inflammatory responses, demonstrating that cytokine activation and peaking occur within the first 24 h [9] and that gene activation decreases by 96 h [28]. In human models, full recovery without treatment is expected within 2–4 weeks [29,30], although the inflammatory peak subsides and signs of skin inflammation, such as visual scoring and transepidermal water loss, decrease in the days following induction [29]. In our SLS-induced ICD model, no significant differences were observed beyond the initial rection regarding mechanical and thermal stimulation. However, visual signs of inflammation and ultrasound analysis continued to progress. In accordance with our findings of uncoupled symptomatology, stimulation, and visual skin inflammation, a study on human volunteers applying higher concentrations of SLS on the arm led to the recovery of functional parameters and visual skin inflammation, while only reactions to chemical skin provocation persisted [29]. It is known that ICD is heterogeneous regarding underlying mechanisms, inflammation processes, time course, and other factors and that it can develop differently based on region and irritant [4,6].

Despite the nociceptive effects of SLS on spontaneous and stimulus-evoked behavior, injections of each pruritogen and the algogen bradykinin elicited more scratching in the controls but not more wiping behavior. This ICD reaction to SLS is different from that caused by the irritant response to the hapten SADBE, which in unsensitized mice led to increased spontaneous scratching and wiping but with little or no allodynia or hyperalgesia to mechanical and thermal stimulations [1]. In contrast, mice that had been sensitized to the hapten in a model of SADBE-induced ACD exhibited greater than normal scratching in reaction to histamine-independent pruritogens and bradykinin [19]. The present finding that ICD from 5% SLS caused increased itching after stimulation with histamine stands in contrast to the effects of SADBE-induced ACD, as well as the dry skin model or atopic dermatitis, and is suggestive of differences between the models in underlying biological mechanisms and potentially different options for treatment [31,32,33,34]. 

It is well established that ICD can co-occur with ACD and atopic dermatitis because these patients may be more susceptible to inflammation and have a lower threshold for developing ICD [35]. This observation suggests a valuable area for further investigation in future animal studies to enhance treatment approaches.

Additionally, our study uncovered differences in reactivity to histamine-dependent and -independent pruritogens which may indicate differences in the involvement of TRPV1 and TRPA1 in downstream signaling, respectively [20,21]. Though histamine has not been demonstrated to have a role in the development of ICD, antihistamines may relieve symptoms and are therefore regarded as a symptomatic treatment option [7]. Similarly, in a murine study, topical antihistamines treated inflammation and improved the skin barrier in ICD [36].

It is known that irritants lead to inflammation initiated by varying mechanisms and mediators. The ICD reactions induced may differ based on the type and concentration of the irritant, the host, the environment, and the application procedure [35]. For example, follicular spongiosis was observed after ICD caused by croton oil, benzalkonium chloride, and dithranol but not SLS [37,38]. Previously, in allergic reactions, increased mRNA expression of different proteins, namely, the chemokine interferon-γ-inducible protein-10 (CXCL10) and the related CXC chemokine receptor 3-activating chemokines (CXCR3) macrophage migration inhibitory factor (MIF) and IP-9, was identified. This was not observed in irritation reactions induced by SLS [39]. Similarly, in our previous study on SADBE-induced ICD, a proinflammatory milieu with cell invasion and increases in IL-1β, TNF-α, and CXCR3 was detected [1]. 

The missing combination of itch and pain and only increased itch after chemical stimulation, as well as the uncoupled sensory symptoms on the first day versus inflammation on the second day, make the 5% SLS model less attractive as a model of pruritic diseases where both itch and pain are present. But the model could be used to study the interaction of itch and pain signaling. Further studies are needed to apply different dosages of SLS and directly compare and contrast itch- and pain-like behaviors for different ICD models. 

Our research highlights the importance of understanding how different irritants elicit distinct inflammatory and sensory pathways, which could inform the structural and immunological characterization of allergenic proteins. The establishment of a standardized model and protocols is paramount for consistent research outcomes. By elucidating the specific sensory and inflammatory mechanisms in ICD, our work contributes to the broader goal of developing targeted treatments for allergic and irritant skin conditions. This model also provides a valuable tool for studying the interplay between itch and pain signaling, offering insights that could lead to better management of pruritic diseases. Therefore, the results of our study can be applied in several critical areas. Investigating whether spontaneous scratching and/or wiping behaviors are heightened in SLS-induced ICD models is essential for understanding symptomatology, refining models for comparative research, distinguishing between skin reaction types, assessing therapeutic strategies, comparing irritants, and guiding clinical applications.

In conclusion, while our study provides valuable insights into the sensory and inflammatory mechanisms of SLS-induced ICD, further refinements are necessary to enhance the model’s fidelity to clinical pruritic conditions. This could involve optimizing dosages, exploring additional sensory endpoints, and incorporating advanced molecular and immunological analyses. Such advancements hold promise for developing more effective therapeutic strategies tailored to the comprehensive treatment of allergic and irritant skin disorders.

## 4. Materials and Methods

### 4.1. Animals

**Figure 6:**
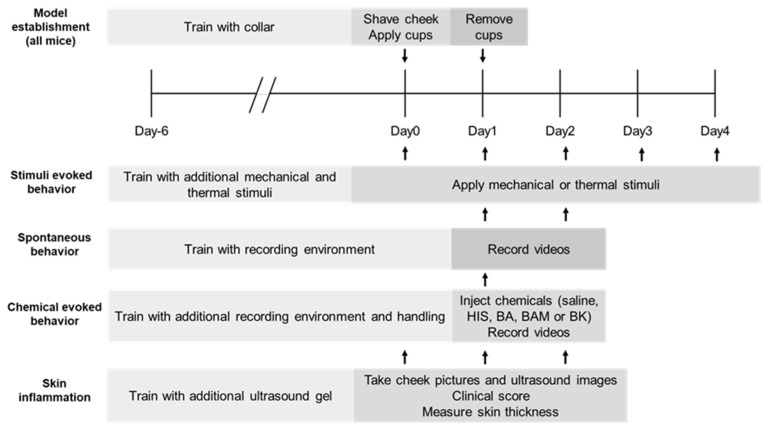
The schematic experimental schedule for the establishment and characterization of the ICD model. For acquisition of ultrasound images, a different group of mice were briefly anesthetized. Arrows symbolize days of testing.

C57/BL6 mice (purchased from Charles River Laboratories), 6–8 weeks old and weighing 20–25 g, were housed in groups of four under a 12 h light/dark cycle with unrestricted access to standard laboratory food and water. Approved by the Institutional Animal Care and Use Committee of Yale University School of Medicine, the experimental procedures adhered to the guidelines of the National Institutes of Health (NIH) and the International Association for the Study of Pain (IASP). Experimenters who were blinded to the experimental conditions conducted and assessed all the experiments. A group size of N = 8–10 per group was aimed for based on previous combined behavioral and morphological aspects of other models, and the same chemicals analyses were applied to determine significant differences. A loss was accounted for in each experimental part in case of illness, cup replacement, or other unforeseen cases; therefore, N = 12 mice per group were included in each experimental part (Figure 6). In the stimulus-evoked experiments, N = 12 mice in the SLS group and N = 12 mice in the control group were tested. For assessment of spontaneous behavior, N = 12 mice in the SLS group and N = 12 mice in the control group were tested. Assessment of chemically evoked behavior was performed on N = 5 × 12 = 60 mice in the SLS group and N = 5 × 12 = 60 mice in the control group. Skin inflammation was assessed for N = 12 mice in the SLS group and N = 12 mice in the control group. Ultrasound imaging was conducted with N = 12 mice in the SLS group and N = 12 mice in the control group before treatment with the vehicle and with N = 10 mice after treatment on days 1 and 2 in the control group. 

### 4.2. Model of Irritant Contact Dermatitis

To induce the SLS ICD model, first, the mice were acclimated to the housing facility for 7 days, followed by 5 days of habituation to handling, the testing environment, and the wearing of an Elizabethan collar around the abdomen. The cheek skin was shaved (1 × 1 cm area) under brief anesthesia with isoflurane (2% in pure oxygen). Two days later, filter paper soaked in 5% SLS (Sigma, St. Louis, MO, USA) in distilled water or the vehicle (distilled water) alone was topically applied to the shaved cheek skin by means of a Finn chamber under brief anesthesia with 2% isoflurane and a flow rate of 300 mL/min of oxygen [22]. Cyanoacrylate was applied as a thin layer on the rim of the chamber to affix the cup to the skin. The Finn chamber (Yale Medical School Machine shop, New Haven, CT, USA) consisted of a polyether ether ketone (PEEK) cup (Trident Plastics, Warminster, PA, USA). The cup was a 8 mm diameter disk with a 1.5 mm wide rim around the edge of the disk and was 0.2–0.3 mm deep and could accommodate the thickness of a 5 mm diameter soaked filter paper. PEEK is a biocompatible plastic used in medical applications in orthopedic surgery and in dental implants, among other uses [40]. 

To prevent the mice from removing the PEEK cup while allowing locomotion, feeding, and drinking, an Elizabethan collar (Harvard Apparatus, Holliston, MA, #NP72-0056) was placed around the abdomen. In the mouse’s home cage, the wire food rack was removed for 24 h and wet chow was placed in a single cup and on the floor to prevent the mouse from being trapped on one side of the cage without access to drinking water. After the PEEK cup was in place for 24 h, the animal was brought back to the laboratory, where the collar, the PEEK cup, and any remaining glue were removed under brief anesthesia [22]. The SLS ICD model was used to simulate acute inflammatory responses in the mice, allowing for the study of skin irritation and subsequent behavioral and physiological changes. This methodology provides a controlled way to induce and analyze inflammatory responses, offering insights into the dynamics of irritant-induced dermatitis.

### 4.3. Behavioral Responses after Mechanical Stimuli and Heat Stimuli 

Mechanical and heat stimuli were delivered to the treated cheek as previously described [1,22] before (day 0) and 1 h (day 1), 24 h (day 2), 48 h (day 3), and 72 h (day 4) after irritant removal. Each mouse was placed in a meshed chamber of 4.5 cm length, 3.5 cm width, and 5 cm height. The mechanical stimuli were delivered using nylon filaments with micrometric (µm) tip diameters that exerted bending forces in millinewtons (mN): specifically, 67 (0.23), 100 (2), 100 (10), and 100 (20). Heat stimuli were administered using a probe comprising a chip resistor (2 × 3 mm) and a thermocouple, delivering either 38 °C for warmth or 52 °C for noxious heat. The stimulus temperature was maintained at the probe–skin interface via electronic circuitry. The duration of stimulus application was 1 s unless it was ended by the animal’s withdrawal. Each stimulus was applied 5 times in ascending order of intensity and then 5 times in descending order. During testing, the mice were video-recorded from the side with a mirror positioned to allow for a two-sided view. Behavioral responses to each stimulus were documented post-stimulation and later verified or corrected upon reviewing the video recordings. The operator conducting the experiments was blinded to the experimental treatment. Each behavioral reaction to a stimulus was categorized by discomfort scores (DSs): “no reaction” (=0); “looking” or turning the head or body towards the stimulus (=1); “withdrawal” by turning the head or body away or pulling backward (=2); rapid “flinching” (=3); “biting” with a quick turn of the head towards the object (=4); “shaking” with a short, rapid body movement (=5); “jumping aside” (=6); “jumping in the air” (=7); and audible squeaking (=8). When observing two immediate behaviors, only the one with the highest score was documented. Each wipe directed at the stimulated cheek was counted and added to the DS. The mean score from ten presentations of the same stimulus was then calculated [22]. Mechanical and heat stimuli were applied to assess changes in sensitivity and pain responses in the treated cheek. This testing helped to determine the extent of sensory alterations, hyperalgesia, and allodynia to mechanical and thermal stimuli, providing quantitative data on the impacts of ICD analyzed by behavioral signs.

### 4.4. Spontaneous Behaviors and Behaviors Evoked by Chemical Injection 

Spontaneous behaviors directed toward the cheek were recorded after 1 h (day 1) and 24 h (day 2) after irritant removal, as described previously [1,17,18]. Each mouse was individually placed in a separate plastic container measuring 9 × 9 × 13 cm^3^, each containing a small amount of bedding. A camcorder positioned above both containers recorded both mice simultaneously. Four mirrors were placed and angled around the container, giving a full view of each mouse. The recording was performed inside a sound-proof room after the mice were placed in the container. After a period of 15 min to allow the mice to acclimate to the recording container, spontaneous behavior was recorded for 1 h. Next, each mouse was taken out of the container and received an intradermal injection into the previously treated cheek (SLS or control) of 5 μL of one of 5 chemicals with a 0.3 mL insulin syringe with a 31-gauge needle, as described previously, e.g., [41]. All injections were conducted within 10 s; then, the mouse was again placed in the container for recording of behavior for 30 min without the experimenter being present in the sound-proof room. Each of the following chemicals were given to a different group of 12 mice: saline (5 µL), histamine (5 μg/5 μL), BAM8-22 (1 μg/5 μL), β-alanine (45 μg/5 μL), and bradykinin (2.65 μg/5 μL).

The video recordings were subsequently analyzed to record the numbers of scratching bouts with the ipsilateral hindlimb as itching sensations and the numbers of wipes with the ipsilateral forelimb directed to the treated cheek as painful sensations. The investigator was kept blinded to the experimental conditions [17]. Spontaneous behaviors and responses to chemical injections were recorded to evaluate itch- and pain-related behaviors. This allowed for a comprehensive understanding of how different stimuli affect behavior and sensory perception in ICD inflamed skin and the altered underlying mechanisms regarding histamine-dependent and -independent itching as well as pain.

### 4.5. Scoring the Severity of Visually Evident Inflammation 

For each experimental group, pictures of the mice’s cheeks were taken in a standardized setup of lighting and camera setting with a color picker and a scale in each picture before (day 0) and 1 h (day 1) and 24 h (day 2) after the irritant removal [1]. At each time point, the erythema and scaling (desquamation) were separately rated to assess the severity of inflammation on the following scale: 0, none; 1, slight; 2, moderate; 3, marked; and 4, very marked [42,43]. Additionally, the cheek skin-fold thickness was measured under brief anesthesia with 2% isoflurane in pure oxygen; the measurements were taken three times using a micrometer (Mitutoyo, Tokyo, Japan), and the means were calculated.

Visual scoring of erythema and scaling, along with measurement of skin-fold thickness, was conducted to quantify the severity of inflammation. These assessments helped correlate visible inflammation with underlying pathological changes, offering a clear metric for evaluating the inflammatory response.

### 4.6. High-Frequency Ultrasound Imaging of Cheek Skin

For the acquisition of ultrasound images, a different group of mice were briefly anesthetized before (day 0) and 1 h (day 1) and 24 h (day 2) after the irritant removal, positioned on a thermoregulated platform. The skin of the cheek was evenly coated with ultrasound gel. Using a high-frequency ultrasound scanner (VisualSonics Vevo 770) in B mode and a 55 MHz transducer (RMV 708), we imaged the superficial skin layers to achieve the optimal spatial resolution. We measured the overall thickness of different skin layers—the stratum corneum, epidermis, dermis, and hypodermis—based on their distinct order, appearance, and echogenicity. Light structures were identified as hyperechoic (white), while darker areas were classified as hypoechoic (gray), using ImageJ. The investigators were kept blinded to the experimental condition [1,44]. High-frequency ultrasound imaging was utilized to measure the thickness of various skin layers, providing detailed structural information. This noninvasive technique allows for precise monitoring of skin changes over time, supporting the evaluation of inflammation and its progression.

### 4.7. Statistical Analysis

Behavioral analyses for each type of spontaneous, site-directed behavior (number of scratching bouts or wipes) involved a mixed-design analysis of variance (ANOVA). This included 2 treatment groups (SLS and control) across 2 testing days with repeated measures (before and 1 h after irritant removal). DS values obtained after stimulation with each vFF and heat stimulus were separately analyzed using a three-way ANOVA: 2 treatment groups (SLS, control) × 4 mechanical stimuli (0.23 mN, 2 mN, 10 mN, and 20 mN) × 2 days post-irritant removal. The effects of force and temperature on responses to mechanical and heat stimuli were analyzed separately using a mixed-design ANOVA: 2 treatment groups (SLS and control) × 4 forces or 2 temperatures × 5 testing days with repeated measures over force or temperature and days of testing. Additionally, erythema score, scaling score, and skin-fold thickness were analyzed with a two-way ANOVA: 2 treatment groups (SLS and control) × 3 testing days with repeated measures. Thickness measurements of different skin layers and power Doppler evaluation of high-frequency ultrasound imaging were analyzed with a one-way ANOVA: 2 treatment groups (SLS and control) × 3 testing days. Bonferroni corrections for family-wise error rates were applied to account for multiple comparisons in each statistical analysis following each ANOVA. A *p*-value < 0.05 was considered statistically significant. The results are presented as means ± SEMs and SDs for categorical variables.

## Data Availability

Data will be made available upon reasonable request directed towards the corresponding authors.
